# Hybrid ABC Optimized MARS-Based Modeling of the Milling Tool Wear from Milling Run Experimental Data

**DOI:** 10.3390/ma9020082

**Published:** 2016-01-28

**Authors:** Paulino José García Nieto, Esperanza García-Gonzalo, Celestino Ordóñez Galán, Antonio Bernardo Sánchez

**Affiliations:** 1 Department of Mathematics, Faculty of Sciences, University of Oviedo, C/Calvo Sotelo s/n, 33007 Oviedo, Spain; lato@orion.ciencias.uniovi.es; 2 Department of Mining Exploitation, University of Oviedo, 33004 Oviedo, Spain; ordonezcelestino@uniovi.es; 3 Department of Mining Technology, Topography and Structures, University of León, 24071 León, Spain; antonio.bernardo@unileon.es

**Keywords:** multivariate adaptive regression splines (MARS), artificial bee colony (ABC), statistical learning techniques, milling tool wear monitoring, hyperparameter selection, regression analysis

## Abstract

Milling cutters are important cutting tools used in milling machines to perform milling operations, which are prone to wear and subsequent failure. In this paper, a practical new hybrid model to predict the milling tool wear in a regular cut, as well as entry cut and exit cut, of a milling tool is proposed. The model was based on the optimization tool termed artificial bee colony (ABC) in combination with multivariate adaptive regression splines (MARS) technique. This optimization mechanism involved the parameter setting in the MARS training procedure, which significantly influences the regression accuracy. Therefore, an ABC–MARS-based model was successfully used here to predict the milling tool flank wear (output variable) as a function of the following input variables: the time duration of experiment, depth of cut, feed, type of material, *etc*. Regression with optimal hyperparameters was performed and a determination coefficient of 0.94 was obtained. The ABC–MARS-based model's goodness of fit to experimental data confirmed the good performance of this model. This new model also allowed us to ascertain the most influential parameters on the milling tool flank wear with a view to proposing milling machine's improvements. Finally, conclusions of this study are exposed.

## 1. Introduction

Machine tools play main roles in modern society. Due to the quick development of the industry and standard of living, it is required more and more that the machine tools have to be safe, reliable, economical, and intelligent. However, several problems may appear, leading to the blockage of the equipment and giving place to serious accidents. In order to avoid accidents, it is mandatory to carry out a scheduled preventive maintenance on the machine tool, which will increase the total operation costs. For this reason, wear failure diagnosis has been accepted completely by the companies since the 1980s. Indeed, machinability can be expressed as the easiness or difficulty in a machining operation involving cutting conditions such as cutting speed, feed rate, and depth of cut. The tool wear describes the gradual failure of cutting tools due to regular operation. Specifically, flank wear is a type of wear in which the portion of the tool in contact with the finished part erodes. Therefore, milling cutters are essential components used in milling machines, which are prone to wear and the subsequent failure. Some general effects of tool wear include increased cutting forces, increased cutting temperatures, poor surface finish, decreased accuracy of finished part, tool breakage, and a change in tool geometry. Therefore, it is very important to be able to predict the wear of this component before failure, preventing serious accidents [1,2,3]. 

Milling is the machining process of using rotary cutters to remove material from a workpiece advancing (or feeding) in a direction at an angle with the axis of the tool. It is one of the most commonly used processes in industry today for machining parts to precise sizes and shapes. After the advent of computer numerical control (CNC), milling machines evolved into machining centers (milling machines with automatic tool changers, tool magazines or carousels, CNC control, coolant systems, and enclosures), generally classified as vertical machining centers (VMCs) and horizontal machining centers (HMCs). Indeed, the milling process removes material by performing many separate, small cuts. Note that a high-quality product often implies a high-quality surface finish and dimensional accuracy. Ideally, a sharp tool should be maintained at all times. A worn tool also results in more friction which, in turn, results in higher cutting temperatures. Unwanted effects may arise from these temperatures, e.g., it may produce untemperedmartensite in heat treatable steel [1,2,3].

Therefore, the motivations behind this study from the technological and economical points of view can be the minimizing of milling's total cost (which can be measured by the total cost of replacing all worn tools during a production period), maximizing of productivity (which can be measured by the total number of parts produced per period), and maximizing of quality of cutting. 

In order to produce quality product, a cutting tool must have three characteristics:

- Hardness: hardness and strength at high temperatures.
- Toughness: so that tools do not chip or fracture.
- Wear resistance: having acceptable tool life before needing to be replaced.

Some materials commonly used in milling tools as cutters are: high-speed steels, carbon tool steels, cast cobalt alloys, cemented carbide, ceramics, *etc*. Therefore, the milling tool wear has to be controlled. In order to estimate the best conditions, *i.e.*, the values of the most influential parameters in milling machines, as well as to predict the milling tool wear, the flank wear was predicted from the other operation parameters [1,2,3,4] by applying an innovative and new regression technique described here. 

**Figure 1:**
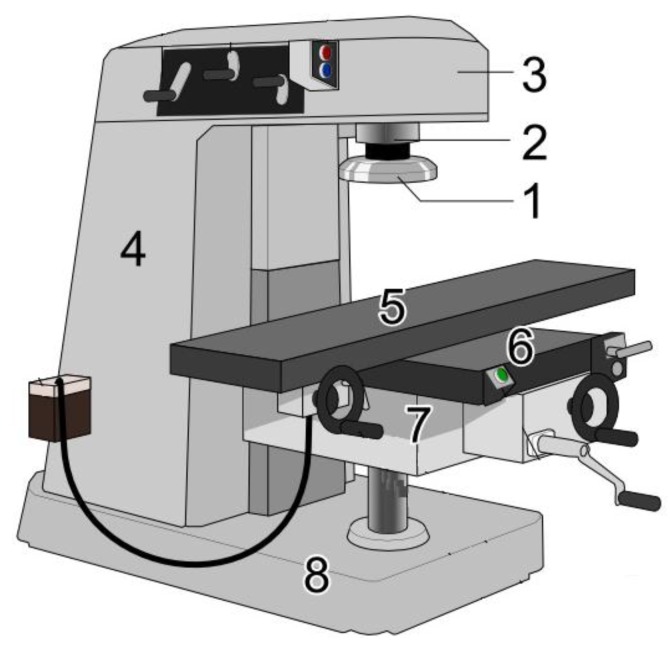
Vertical milling machine: (1) milling cutter; (2) spindle; (3) top slide or over arm; (4) column; (5) table; (6) Y-axis slide; (7) knee; and (8) base.

Indeed, the main goal of this research work was to obtain the dependence relationship of the milling tool wear (output variable) as a function of the ten milling operation input variables [1,2,3] indicated later. Different methods have been used previously to tackle this kind of problem, such as linear regression [5,6], neural networks [6,7,8,9,10], support vector machines [11,12], genetic programming [13,14,15,16], and so on. The objective of this study is to evaluate the application of multivariate adaptive regression splines (MARS) in combination with the artificial bee colony (ABC) optimization technique to identify the milling tool flank wear (see Figure 1). On the one hand, the MARS technique is based on the statistical learning theory and is a new class of model that can be used to predict values in very different areas [17,18,19,20,21]. It is a non-parametric regression technique and can be seen as an extension of linear models that automatically models nonlinearities and complex interactions between variables. Some motivations behind the application of the proposed method with respect to other already existing techniques are as follows: (1) MARS models are more flexible than linear regression models; (2) MARS models are simple to understand and interpret; (3) MARS can handle both continuous and categorical data; (4) MARS models tend to have a good bias-variance trade-off; and (5) MARS models give us an explicit mathematical formula of the dependent variable as a function of the independent variables through an expansion of basis functions (hinge functions and products of two or more hinge functions). This last feature is a fundamental difference compared to other alternative methods because most of them behave like a black box. On the other hand, the artificial bee colony (ABC) technique is an optimization algorithm based on the intelligent foraging behavior of honey bee swarm [22,23,24]. The only limitation of this model is due to its special feature being a data-driven technique.

In summary, this paper is organized as follows: firstly, Section 2 describes the materials, methods and dataset; then, Section 3 presents and discusses the results of the new hybrid ABC–SVM-based method; and finally, Section 4 presents the main conclusions of this research work.

## 2. Materials and Methods

### 2.1. Experimental Datasets

The dataset represents experiments from runs on a milling machine under various operating conditions (see Appendix A). In particular, tool wear was investigated here in a regular cut, as well as an entry cut and an exit cut. Data sampled by three different types of sensors (acoustic emission sensor, vibration sensor, current sensor) were acquired at several positions [1,2,3,4]. The data is organized in a MATLAB structure array [25] with fields as shown in Table 1 below:

**Table 1:** Structure field names and description.

Field Name	Description
case	Case number (1–16)
run	Counter for experimental runs in each case
VB (mm)	Flank wear, measured after runs; Measurements for VB were not taken after each run
Time (mm)	Duration of experiment (restarts for each case)
DOC (mm)	Depth of cut (does not vary for each case)
Feed (mm/rev)	Feed (does not vary for each case)
Material	Material (does not vary for each case)
smcAC	AC spindle motor current
smcDC	DC spindle motor current
vib_table	Table vibration
vib_spindle	Spindle vibration
AE_table	Acoustic emission at table
AE_spindle	Acoustic emission at spindle

There are 16 cases with a varying number of runs. The number of runs was dependent on the degree of flank wear that was measured between runs at irregular intervals up to a wear limit (and sometimes beyond). Flank wear was not always measured and at times when no measurements were taken, no entry was made. The sixteen cases are enumerated in Table 2.

**Table 2:** Experimental conditions.

Case	Depth of Cut (mm)	Feed (mm/rev)	Workpiece Material
1	1.5	0.5	cast iron
2	0.75	0.5	cast iron
3	0.75	0.25	cast iron
4	1.5	0.25	cast iron
5	1.5	0.5	steel
6	1.5	0.25	steel
7	0.75	0.25	steel
8	0.75	0.5	steel
9	1.5	0.5	cast iron
10	1.5	0.25	cast iron
11	0.75	0.25	cast iron
12	0.75	0.5	cast iron
13	0.75	0.25	steel
14	0.75	0.5	steel
15	1.5	0.25	steel
16	1.5	0.5	steel

**Figure 2:**
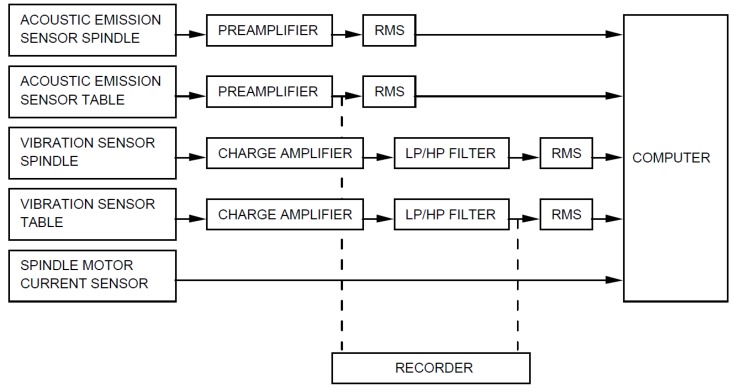
Experimental setup.

The setup of the experiment is as depicted in Figure 2 below. The basic setup encompasses the spindle and the table of the Matsuura machining center MC-510V. An acoustic emission sensor and a vibration sensor are each mounted to the table and the spindle of the machining center. The signals from all sensors are amplified and filtered, then fed through two root mean square (RMS) converters before they enter the computer for data acquisition. The signal from a spindle motor current sensor is fed into the computer without further processing. The matrix for the parameters chosen for the experiments were guided by industrial applicability and recommended manufacturer’s settings. Therefore, the cutting speed was set to 200 m/min which is equivalent to 826 rev/min. Two different depths of cut were chosen, 1.5 mm and 0.75 mm.

Additionally, two feeds were taken, 0.5 mm/rev and 0.25 mm/rev which translate into 413 mm/min and 206.5 mm/min, respectively. Two types of workpiece material, cast iron and stainless steel J45, were used. Furthermore, a 70 mm face mill with sixinserts KC710 was chosen as the cutting tool. The insert KC710 is coated with multiple layers of titanium carbide, titanium carbon nitride, and titanium nitride (TiC/TiC-N/TiN) in sequence. These layers retain the toughness of tungsten carbide but have improved resistance to cratering and edge wear. At the same time, they have the advantage of titanium carbide plus reduced face friction. These choices equal eightdifferent settings. All experiments were done a second time with the same parameters with a second set of inserts. The size of the workpieces was 483 × 178 × 51 mm.

#### 2.1.1. Data Acquisition and Processing

As described in the previous section, the data were sent through a high speed data acquisition board with maximal sampling rate of 100 kHz. The sampled output of the data was used for the signal processing software. LabVIEW^®^ [26] was used for this task. This software is a general purpose programming development system which uses a graphical language (G). With G, programs are created in block diagram form. The chosen layout has allowed for data acquisition, storage, presentation, and processing. Data were stored to allow for real time simulation, and also later analysis.

Several sensor signals underwent preprocessing. In most cases, the signal was amplified to be able to meet threshold requirements of equipment. In particular, the signals from the acoustic emission sensors and from the vibration sensors were amplified to be in the range of ±5 V for maximum load, considering the maximum allowable range of the equipment. The signals were filtered by a high-pass filter, the vibration sensor signals were additionally filtered with a low-pass filter. Corner frequencies were chosen according to the noise that could be observed on an oscilloscope. Periodical noise of 180 Hz was observed on the oscilloscope for the vibration signal corresponding to the third harmonic of the main power supply. Therefore, the chosen corner frequency for the low-pass filter was 400 Hz. For the high-pass filter, 1 kHz was chosen. Above 8 kHz, the range of the acoustic emission sensor ends. That is, readings above that frequency cannot be attributed to any occurrence in the machining process. Since it clutters the signal unnecessarily, it was filtered out. Acoustic emission and vibration signals were fed through an RMS device. Its use smoothes the signal and makes it more accessible to signal processing. The root mean square (RMS) is a statistical measure of the magnitude of a varying quantity and it is proportional to the energy contents of the signal. The RMS of a function *f* for a period of time is defined by [1,2,3,4]:

(1) $$RMS=\sqrt{\frac{1}{\Delta T}\int_{0}^{\Delta T}f^{2}\left(t\right)\;dt}$$

where ΔT is time constant and f(t) the signal function. In this case, as the data is discrete the formula used was [1,2,3,4]:

(2) $$RMS=\sqrt{\frac{1}{n}\sum_{k=1}^{n}{\left(f\left(t_{k}\right)\right)}^{2}}$$

where*n* is the number of samples. There are only a value of the variables *case*, *run*, *VB*, and *time* per each structure array in the dataset. Furthermore, variables *DOC*, *Feed*, and *Material* do not vary within each case. Additionally, variables *smcAC, smcDC, vib_table, vib_spindle, AE_table*, and *AE_spindle* are curves with 9000 points each. The *RMS* has been obtained for each of these curves in order to have only one value per each one of the other variable values.

#### 2.1.2. Tool Wear

In an industrial process, the manufacturing of a high-quality product often involves a high-quality surface finish and dimensional accuracy. Therefore, a sharp tool must be kept at all times. A cutting tool in disrepair deforms the surface to a greater depth and may tear the surface which, in turn, may lower the fatigue resistance. Additionally, a cutting tool with considerable wear also results in more friction which in turn results in higher cutting temperatures. Indeed, undesirable effects may occur from these temperatures such as the presence of untemperedmartensite in heat treatable steel. Therefore, tool wear has to be controlled [1,2,3,4].

**Figure 3:**
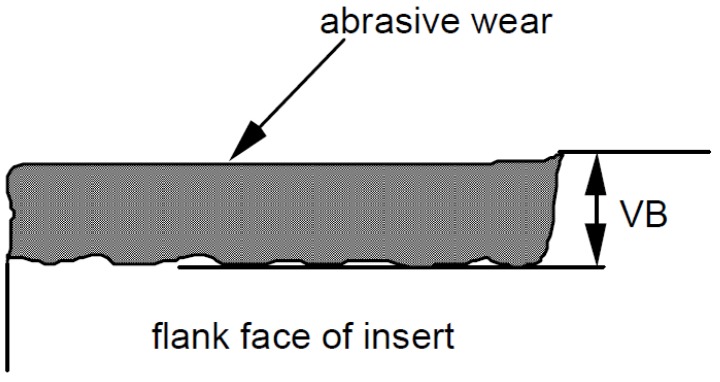
Tool wear VB as it is seen on the insert.

Tool wear comes in different forms. Apart from the intuitive rounding of the cutting edge, crater wear on the rake face due to the abrasion of the sliding of the chip on the rake face and flank wear due to friction of the tool on the workpiece occur [27,28]. Speed of cutting, more than other parameters, influence the rate of wear; depth of cut and feed rate also affect the tool life. In our experiments, we measured the *flank wear* VB as a generally-accepted parameter for evaluating tool wear (see Figure 3) [1,2,3,4,27,28,29].

The flank wear VB is measured as the distance from the cutting edge to the end of the abrasive wear on the flank face of the tool. The flank wear was observed during the experiments. The insert was taken out of the tool and the wear was measured with the help of a microscope.

### 2.2. Multivariate Adaptive Regression Spline (MARS)

**Figure 4:**
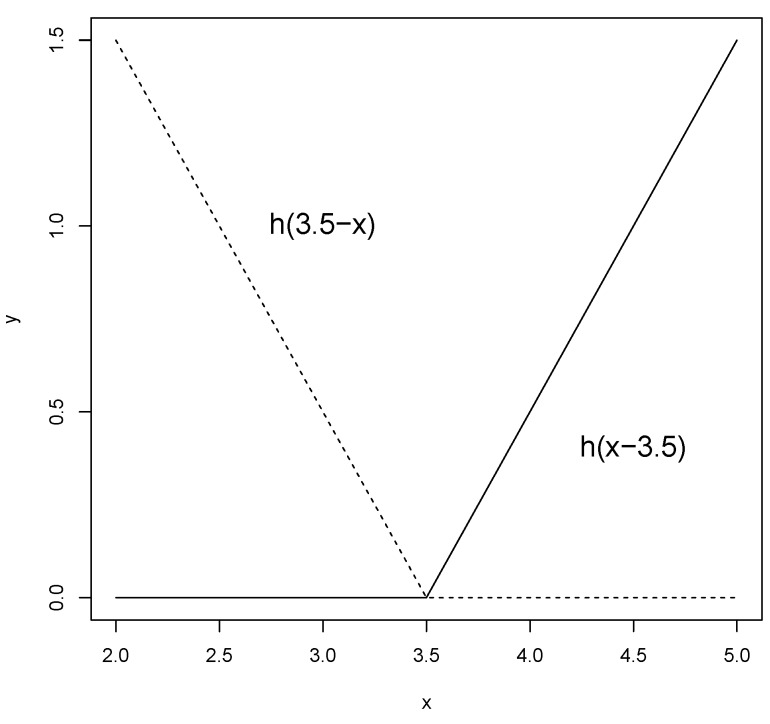
A graphical representation of a spline basis function. The left spline (x<t, −(x−t)) is shown as a dashed line and the right spline (x>t, +(x−t)) as a solid line.

Multivariate adaptive regression splines (MARS) is a multivariate nonparametric classification/regression technique [17,18,19,20,21]. Its main purpose is to predict the values of a continuous dependent variable, y(n×1), from a set of independent explanatory variables, X(n×p). The MARS model can be represented as:

(3) y=f(X)+e

where*f* is a weighted sum of basis functions that depend on X and e is an error vector of dimension (n×1). MARS can be considered as a generalization of *classification and regression trees* (CART) [20] and is able to overcome some limitations of CART. MARS model does not require any a priori assumptions about the underlying functional relationship between dependent and independent variables. Instead, this relation is uncovered from a set of coefficients and piecewise polynomials of degree *q* (basis functions) that are entirely *driven* from the regression data (X,y). The MARS regression model is constructed by fitting basis functions to distinct intervals of the independent variables. Generally, piecewise polynomials, also called splines, have pieces smoothly connected together. In MARS terminology, the joining points of the polynomials are called knots, nodes, or breakdown points. These will be denoted by the small letter t. For a spline of degree q each segment is a polynomial function. MARS uses two-sided truncated power functions as spline basis functions, described by the following equations [17,18,19,20,21]:

(4) $${\left[-\left(x-t\right)\right]}_{+}^{q}=\{\begin{array}{cc} {\left(t-x\right)}^{q} & if\;x<t\\ 0 & otherwise \end{array}$$

(5) $${\left[+\left(x-t\right)\right]}_{+}^{q}=\{\begin{array}{cc} {\left(t-x\right)}^{q} & if\;x\geq t\\ 0 & otherwise \end{array}$$

where q (≥0) is the power to which the splines are raised and which determines the degree of smoothness of the resultant function estimate. When q=1, which is the case in this study, only simple linear splines are considered. A pair of splines for q=1 at the knot t=3.5 is presented in Figure 4.

The MARS model of a dependent variable y with *M* basis functions (terms) can be written as [30,31,32,33,34,35,36]:

(6) $$\hat{y}={\hat{f}}_{M}\left(x\right)=c_{0}+\sum_{m=1}^{M}c_{m}B_{m}\left(x\right)$$

where $\hat{y}$ is the dependent variable predicted by the MARS model, $c_{0}$ is a constant, $B_{m}\left(x\right)$ is the *m*-th basis function, which may be a single spline basis functions, and $c_{m}$ is the coefficient of the *m*-th basis functions. Both variables to be introduced into the model and the knot positions for each individual variable have to be optimized. For a data set X containing *n* objects and *p* explanatory variables, there are N=n×p pairs of spline basis functions, given by Equations (4) and (5), with knot locations $x_{ij}$(i=1, 2,...,n;  j=1, 2,...,p).

A two-step procedure is followed to construct the final model. First, in order to select the consecutive pairs of basis functions of the model, a two-at-a-time forward stepwise procedure is implemented [17,18,19,20,21]. This forward stepwise selection of basis functions leads to a very complex and overfitted model. Such a model, although it fits the data well, has poor predictive abilities for new objects. To improve the prediction, the redundant basis functions are removed one at a time using a backward stepwise procedure. To determine which basis functions should be included in the model, MARS utilizes the generalized cross-validation (*GCV*) [30,31,32,33,34,35,36]. In this way, the *GCV* is the mean squared residual error divided by a penalty dependent on the model complexity. The *GCV* criterion is defined in the following way [17,18,19,20,21,33,34,35,36]:

(7) $$GCV\left(M\right)=\frac{\frac{1}{n}\sum_{i=1}^{n}{\left(y_{i}-{\hat{f}}_{M}\left(x_{i}\right)\right)}^{2}}{{\left(1-C\left(M\right)/n\right)}^{2}}$$

where C(M) is a complexity penalty that increases with the number of basis functions in the model and which is defined as [30,31,32,33,34,35,36]:

(8) C(M)=(M+1)+d M

where*M* is the number of basis functions in Equation (6), and the parameter *d* is a penalty for each basis function included into the model. It can be also regarded as a smoothing parameter. Large values of *d* lead to fewer basis functions and therefore smoother function estimates.

Once the MARS model is constructed, it is possible to evaluate the importance of the explanatory variables used to construct the basis functions. Establishing predictor importance is in general a complex problem which in general requires the use of more than one criterion. In order to obtain reliable results, it is convenient the use of the *GCV* parameter explained before together with the parameters Nsubsets (criterion counts the number of model subsets in which each variable is included) and the residual sum of squares *RSS* [17,18,19,20,21,33,34,35,36].

### 2.3. The Artificial Bee Colony (ABC) Algorithm

The algorithmArtificial Bee Colony (ABC) is an evolutionary optimization algorithm inspired in the behavior of bees foraging food sources [22,23,37]. In the evolutionary algorithms, a population of possible solutions evolves with the iterations toward the optimum using a strategy that involves some random component [22,23,37]. The ABC is also in the group of swarm intelligence algorithms that is characterized by the sharing of information between the individuals in the swarm or population. Indeed, in the ABC technique, the colony consists of three groups of bees: employed bees, onlookers, and scouts. It is assumed that there is only one artificial employed bee for each food source. In other words, the number of employed bees in the colony is equal to the number of food sources around the hive. After going to their food source, employed bees come back to the hive and they dance. If a food source has been abandoned, the employed becomes a scout and starts to search for a new food source. Onlookers observe the dances and proceed to choose food sources following the indications of the dances. Therefore, ABC considers three phases [22,23,24,37]:

- The employed bee phase: each food source is foraged by employed bees.
- The onlooker bee phase: they choose a food source watching the dance of employed bees within the hive. The foraging is supervised and sometimes corrected by the onlooker.
- The scout bee phase: the depleted sources are discarded and the scout bees search for new food sources.

**Figure 5:**
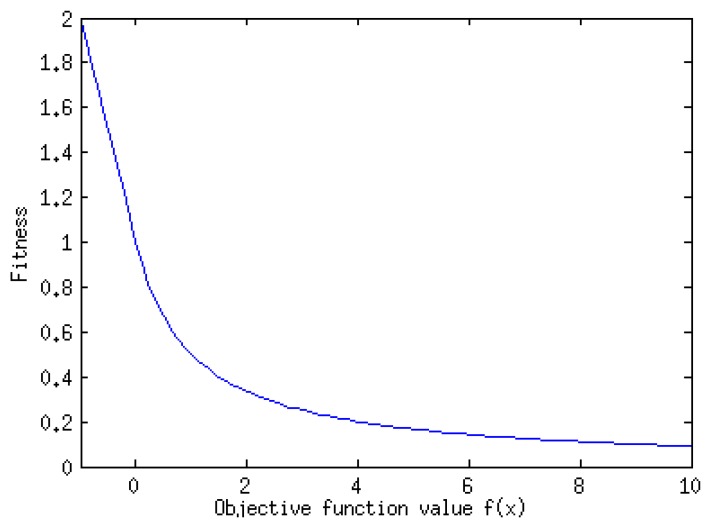
Relation between the fitness of a food source and objective function.

The *N* food sources are the possible set of solutions and are represented by the vectors $p_{i}$. It represents its position in the search space of possible solutions. The food source dimension is the number of parameters of the optimization problem. The algorithm initializes the food sources or possible solutions of the problem randomly in a plausible hypercube and the fitness of each food source is evaluated. The relation between the objective function *f* and the fitness of a food source is given by (see Figure 5):

(9) $$Fitness\left(F\left(p_{i}\right)\right)=\{\begin{array}{ccc} \frac{1}{1+F\left(p_{i}\right)} & if & F\left(p_{i}\right)>0\\ 1+\left|F\left(p_{i}\right)\right| & if & F\left(p_{i}\right)\leq 0 \end{array}\;i=1,...,N$$

The lower the objective function value, the higher the fitness. As the algorithm searches for the highest fitness of a food source, it searches a minimum for the objective function. If we want to maximize a function *g,* the objective function, must be F=−g and then a maximum of *g* is a minimum of *F*.

#### 2.3.1. The Employed Bee Phase

In the first phase the employed bees forage the food sources and tryto introduce a variation of every *i* food source according to the equation [22,23,24,37]:

(10) $$v_{ij}=p_{ij}+R_{ij}\left(p_{kj}-p_{ij}\right)$$

where*j* is the randomly chosen parameter we are modifying, *k* a randomly chosen food source different from *i,* and *R_ij_* a number chosen randomly in [−1, 1]. Once calculated $v_{ij}$, its fitness is obtained. If this is higher than $fitness\left(F\left(p_{ij}\right)\right)$, its value is changed to $v_{ij}$ and the trial counter set to one. If not, the value of the food source does not change and the trial counter is increased.

#### 2.3.2. The Onlooker Bee Phase

For each food source $p_{i}$, we draw a number $r_{i}$ in [0,  1]. If $r_{i}<prob_{i}$, we try again to change one parameter in the food source. The quantity $prob_{i}$ is obtained from the fitness of this food source as follows [22,23,24,37]:

(11) $$prob_{i}=\frac{0.9\;Fitness\left(F\left(p_{i}\right)\right)}{\underset{k=1,...,N}{\max}\left(Fitness\left(F\left(p_{k}\right)\right)\right)}+0.1$$

#### 2.3.3. The Scout Bee Phase

If after a determined number of trials a food source is not improved, it is discarded and a new one is randomly chosen from the initial search space. The food source with the highest fitness is the temporal optimum in this iteration [22,23,24,37].

This cycle is continued until a stopping criterion is met. In the present case, the stopping condition has been a maximum number of iterations and the repetition of the optimum for a determined number of iterations. If this occurs, it is assumed that the algorithm has already converged.

### 2.4. The Goodness-of-Fit of This Approach

The operation input variables considered in this research work are shown in Table 3 [1,2,3,4]. Therefore, the total number of predicting variables used to build the hybrid ABC–MARS-based model was nine. The output predicted variable is the flank wear (VB) measured in mm. The VB missing values have been removed and 145 samples remain (see Appendix A). Furthermore, the input variable *material* is a categorical variable.

**Table 3:** Operation input variables used in this study and their names.

Input Variables	Name of the Variable
Time (mm)	Time
Depth of cut (mm)	DOC
Feed (mm/rev)	Feed
Material	Material
AC spindle motor current	smcAC
Table vibration	vib_table
Spindle vibration	vib_spindle
Acoustic emission at table	AE_table
Acoustic emission at spindle	AE_spindle

To estimate flank wear (VB) from other operation parameters it is important to select the model that best fits the experimental data [27,28,29,38,39]. To measure the goodness-of-fit the criterion considered was the coefficient of determination $R^{2}$ [38,39]. This ratio indicates the proportion of total variation in the dependent variable explained by the model (flank wear in our case). A dataset takes values $t_{i}$, each of which has an associated modelled value $y_{i}$. The former are called the observed values and the latter are often referred to as the predicted values. Variability in the dataset is measured through different sums of squares [38,39]:

- $SS_{tot}=\sum_{i=1}^{n}{\left(t_{i}-\bar{t}\right)}^{2}$: the total sum of squares, proportional to the sample variance.
- $SS_{reg}=\sum_{i=1}^{n}{\left(y_{i}-\bar{t}\right)}^{2}$: the regression sum of squares, also called the explained sum of squares.
- $SS_{err}=\sum_{i=1}^{n}{\left(t_{i}-y_{i}\right)}^{2}$: the residual sum of squares.

In the previous sums, $\bar{t}$ is the mean of the *n* observed data:

(12) $$\bar{t}=\frac{1}{n}\sum_{i=1}^{n}t_{i}$$

Bearing in mind the above sums, the general definition of the coefficient of determination is:

(13) $$R^{2}\equiv 1-\frac{SS_{err}}{SS_{tot}}$$

A coefficient of determination value of 1.0 indicates that the regression curve fits the data perfectly.

Furthermore, it is well known that the MARS technique is strongly dependent on the following hyperparameters [17,18,19,20,21,33,34,35,36]:

- Maximum number of basis functions (Maxfuncs): maximum number of model terms before pruning, *i.e.*, the maximum number of terms created by the forward pass.
- Penalty parameter (*d*): is the Generalized Cross Validation (GCV) penalty per knot. A value of 0 penalizes only terms, not knots. The value −1 means no penalty.
- Interactions: maximum degree of interaction between variables.

Some methods often used to determine suitable hyperparameters are [17,18,19,20,21,33,34,35,36]: grid search, random search, Nelder-Mead search, heuristic search, genetic algorithms, pattern search, *etc*. In this research work, the artificial bee volony (ABC) technique was applied [22,23,24,37].

**Figure 6:**
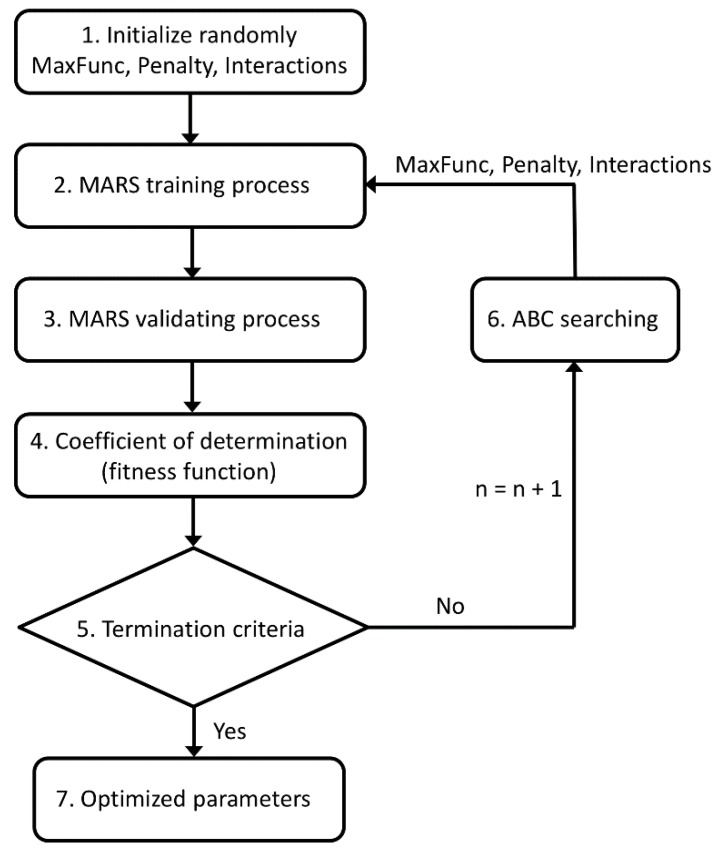
Flowchart of the new hybrid ABC–MARS-based model.

To fix ideas, a novel hybrid ABC-MARS-based model was applied to predict the milling tool wear (output variable) from the other nine remaining variables (input variables) in a milling process [1,2,3,4], studying their influence in order to optimize its calculation through the analysis of the coefficient of determination $R^{2}$ with success. Figure 6 shows the flowchart of this new hybrid ABC-MARS-based model developed in this study.

Cross-validation was the standard technique used here for finding the real coefficient ofdetermination ($R^{2}$) [38,39,40]. Indeed, in order to guarantee the prediction ability of the ABC-MARS-based model, an exhaustive 10-fold cross-validation algorithm was used [40]. The referred algorithm consists in splitting the sample into 10 parts and using nineof them for training and the remaining one for testing. This process was performed 10 times using each of the parties of the 10 divisions for testing and calculating the average error. Therefore, all the possible variability of ABC-MARS-based model parameters has been evaluated in order to get the optimum point, looking for those parameters that minimize the average error. 

The regression modeling has been performed with multivariate adaptive regression splines (MARS) method, using the Earth library [41] together with the ABC technique with the ABCOptim package [42] from the R Project. The bounds (initial ranges) of the space of solutions used in ABC technique are shown in Table 4. Twenty bees and ten food sources have been used in the ABC optimization. The stopping criteria have been 20 iterations with unchanged results of the coefficient of determination $R^{2}$ along with a maximum number of 500 iterations. The problem was solved in a computer with a Intel(R) Core(TM) i7-4770 CPU @ 3.40GHz with 7.7 GB of RAM and Ubuntu 14.04 LTS operating system.

**Table 4:** Initial ranges of the three hyperparameters of the ABC–MARS-based model fitted in this study.

MARS Hyperparameters	Lower Limit	Upper Limit
Maximum number of basis functions (MaxFuncs)	3	200
Penalty parameter (*d*)	−1	4
Interactions	1	6

To optimize the MARS parameters, the ABC module is used. The ABC searches for the best Maxfuncs, Penalty, and Interactions parameters by comparing the cross-validation error in every iteration. The search space is organized in three dimensions, one for each parameter. The main fitness factor or objective function is the coefficient of determination ($R^{2}$). 

## 3. Analysis of Results and Discussion

Table 5 shows the optimal hyperparameters of the best fitted ABC–MARS-based model found with the artificial bee colony (ABC) technique.

**Table 5:** Optimal hyperparameters of the best fitted MARS model found with the ABC technique.

Hyperparameters	Optimal Values
MaxFuncs	129
Penalty (*d*)	3
Interactions	2

The results of the best fitted ABC–MARS-based model computed using all the available data observations are shown in Table 6. Table 6 shows a list of 23 main basis functions for fitted ABC-MARS-based model and their coefficients, respectively. Please note that h(x)=x if x>0 and h(x)=0 if x≤0. Therefore, the MARS model is a form of nonparametric regression technique and can be seen as an extension of linear models that automatically models nonlinearities and interactions asa weighted sum of basis functions called *hinge functions* [30,31,32,33,34,35,36].

**Table 6:** List of basis functions of the best fitted ABC–MARS–based model for the tool flank wear (VB) and their coefficients $c_{i}$ .

*B*_i_	Definition	$c_{i}$
*B*_1_	1	−0.0591
*B*_2_	DOC	2.1405
*B*_3_	Feed	0.1739
*B*_4_	Material2	0.6330
*B*_5_	*h*(smcAC − 1.9747)	−0.3452
*B*_6_	*h*(2.4182 − smcAC)	−1.7527
*B*_7_	*h*(smcAC − 2.4182)	3.0985
*B*_8_	*h*(9 − Time)×DOC	−0.0139
*B*_9_	*h*(Time − 9) ×DOC	0.0100
*B*_10_	*h*(34 − Time)×Material2	−0.0137
*B*_11_	*h*(Time − 34)×Material2	0.0514
*B*_12_	*h*(smcAC − 1.4807)×DOC	−2.2377
*B*_13_	*h*(1.4807 − smcAC)×DOC	2.5430
*B*_14_	*h*(smcAC − 2.1822)×Feed	0.9637
*B*_15_	*h*(0.3161 − vib_spindle)Feed	6.7364
*B*_16_	*h*(0.2111 − AE_spindle)×Material2	−2.3683
*B*_17_	*h*(AE_spindle − 0.2111)×Material2	0.9884
*B*_18_	*h*(51 − Time)×*h*(2.4182 − smcAC)	0.0014
*B*_19_	*h*(DOC − 0.75)×*h*(2.4182 − smcAC)	−2.3128
*B*_20_	*h*(smcAC − 2.4182)×*h*(vib_table − 1.1543)	5.4917
*B*_21_	*h*(smcAC − 2.4182)×*h*(1.1543 − vib_table)	0.6416
*B*_22_	*h*(2.4182 − smcAC)×*h*(0.3329 − vib_table)	−1.4002
*B*_23_	*h*(smcAC − 2.4182)×*h*(vib_table − 0.2976)	−3.3972

**Figure 7:**
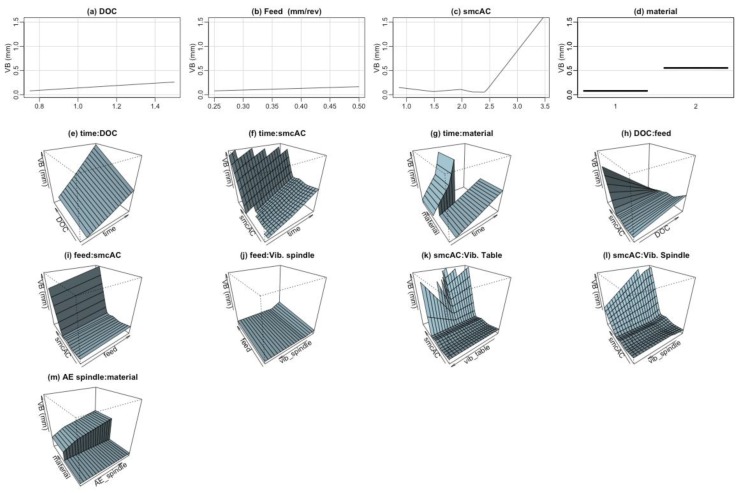
Graphical representation of the terms that constitute the MARS model for the tool flank wear (VB): (**a**) first order term of the predictor variable Depth of cut (DOC); (**b**) first order term of the predictor variable Feed; (**c**) first order term of the predictor variable AC spindle motor current (smcAC); (**d**) first order term of the predictor variable Material; (**e**) second order term of the predictor variables Time and Depth of cut; (**f**) second order term of the predictor variables Time and AC spindle motor current (smcAC); (**g**) second order term of the predictor variables Time and Material; (**h**) second order term of the predictor variables Depth of cut (DOC) and Feed; (**i**) second order term of the predictor variables Feed and AC spindle motor current (smcAC); (**j**) second order term of the predictor variables Feed and Spindle vibration (vib_spindle); (**k**) second order term of the predictor variables AC spindle motor current (smcAC) and Table vibration (vib_table); (**l**) second order term of the predictor variables AC spindle motor current (smcAC) and Spindle vibration (vib_spindle); and (**m**) second order term of the predictor variables Acoustic emission at spindle (AE_spindle) and Material.

Furthermore, a graphical representation of the terms that constitute the best fitted ABC-MARS-based model for the flank wear (VB) can be seen in Figure 7.

Additionally, Table 7 shows the determination and correlation coefficients for the ABC-MARS-based model. An important goodness of fit, that is to say, a good agreement between the model and the experimental data can be inferred from these results.

**Table 7:** Coefficient of determination ($R^{2}$) and correlation coefficient for the hybrid ABC-MARS-based model fitted in this study.

Hybrid Model	Coefficient of Determination ($R^{2}$)/Correlation Coefficient (*r*)
*ABC–MARS*	0.94/0.97

**Figure 8:**
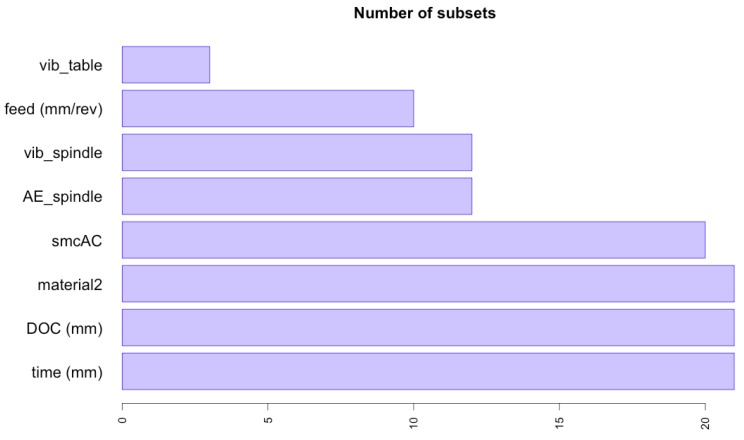
Relative importance of the input operation variables to predict the tool flank wear (VB) in the fitted ABC-MARS-based model.

The significance ranking for the nine input variables predicting the tool flank wear (output variable) in this high nonlinear complex problem is shown in Table 8 and Figure 8. Thus, for the MARS model the most significant variables in the flank wear prediction are the time, depth of cut, and material followed by AC spindle motor current, acoustic emission at spindle, spindle vibration, feed, and finally table vibration. This model considers that acoustic emission at table values have no influence in the flank wear prediction.

**Table 8:** Significance ranking for the variables involved in the best fitted ABC-MARS-based model for the tool flank wear (VB) prediction according to criteria Nsubsets, *GCV,* and *RSS*.

Input Variable	Nsubsets	*GCV*	*RSS*
Time	21	95.9	94.2
Depth of cut (DOC)	21	95.9	94.2
Material2 (Steel)	21	95.9	94.2
AC spindle motor current (smcAC)	20	100.0	100.0
Acoustic emission at spindle (AE_spindle)	12	27.7	27.1
Spindle vibration (vib_spindle)	12	21.6	22.5
Feed	10	18.6	19.3
Table vibration (vib_table)	3	6.8	8.1

**Figure 9:**
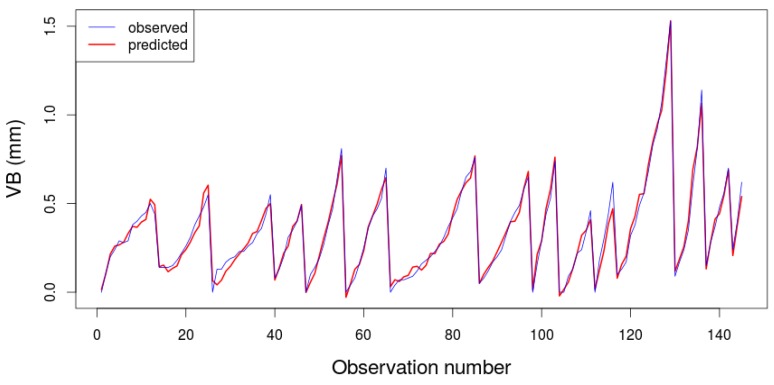
Comparison between the flank wear (VB) in mmmeasured experimentally and flank wear predicted by the ABC–MARS-based model in the milling process using the optimal hyperparameters $\left(R^{2}=0.94\right)$ .

Finally, this research work was able to predict the milling tool flank wear in agreement to the actual milling tool wear values observed experimentally using this hybrid ABC-MARS-based model with great accurateness and success. Indeed, Figure 9 shows the comparison between the flank wear (VB) in mm measured and flank wear predicted by the ABC–MARS-based model in the milling process using the optimal hyperparameters calculated previously indicated in Table 5.

## 4. Conclusions

Based on the experimental and numerical results, the main findings of this research work can be summarized as follows:

- Firstly, the hypothesis that the milling tool flank wear can be accurately modeled by using a hybrid ABC–MARS-based model in the industrial milling process was confirmed.
- Secondly, a high coefficient of determination equal to 0.94 was obtained when this hybrid ABC–MARS-based model was applied to the experimental dataset. Indeed, the predicted results for this model have been proved to be consistent with the historical dataset of observed actual milling tool wear values (see Figure 9).
- Thirdly, the significance order of the input variables involved in the prediction of the milling tool flank wear was set. This is one of the main findings in this research work. Specifically, the duration of experiment (Time), Deep of cut (DOC), and Material2 (steel) variables could be considered the most influential parameters in the prediction of milling tool flank wear in the same proportion, followed by AC spindle motor current (smcAC), respectively.
- Finally, the results verify that the hybrid ABC–MARS-based regression method significantly improves the generalization capability achievable with only the MARS-based regressor. Additionally, this hybrid model is a completely generic since its application can be extended similarly to other processes such as turning, drilling, grinding, *etc*. Indeed, the extendibility of the achieved results to other technological situations and machines for exploitation in a real industrial context is immediate following the methodology previously indicated in this study.

In summary, authors of this research work have confidence that the results obtained in this study will be useful to promote new future research works in this direction.

## Supplementary Materials

Supplementary dataset related to this article can be found at https://dl.dropboxusercontent.com/u/36679320/Milling_dataset.xls.
